# Sampson's Artery Hemorrhage after Inguinal Hernia Repair: Second Case Reported

**DOI:** 10.1155/2016/2534037

**Published:** 2016-05-10

**Authors:** Joseph Adjei Boachie, Eduardo Smith-Singares

**Affiliations:** ^1^College of Medicine, University of Illinois at Chicago, Chicago, IL 60612, USA; ^2^Department of Surgery, Division of Surgical Critical Care, University of Illinois at Chicago, Chicago, IL 60612, USA

## Abstract

Sampson artery is normally obliterated in postembryonic development. In rare cases it can remain patent and complicate a routine outpatient herniorrhaphy when severed. This is the second reported case in the available English literature of hemoperitoneum due to bleeding from a patent Sampson's artery following an open inguinal hernia repair.

## 1. Introduction

Rarely do complications occur on a routine inguinal hernia repair. It is considered to be one of the safest operative procedures and one of the most common. The repair can be performed using either a laparoscopic or open technique. The laparoscopic technique has the benefit of less pain and a decreased risk for infection; it is also more cosmetically pleasing and offers a quicker return to regular activities [1]. In a recent comparison, the two-year recurrence rate for both techniques was similar [2]. Likewise, the complication rate for both techniques is comparable. Few rare complications, as in this case report, may prevent the use of one technique over the other. This report is a case of hemoperitoneum following an uncomplicated inguinal hernia repair in a female patient. The source was traced to a bleeding segment of the artery of the round ligament of the uterus, also known as the Artery of Sampson. This is the second case of such complication reported in the available English literature. Buch et al. reported the first case of such complication following an uncomplicated inguinal hernia repair [3].

## 2. Case Report

**Figure 1:**
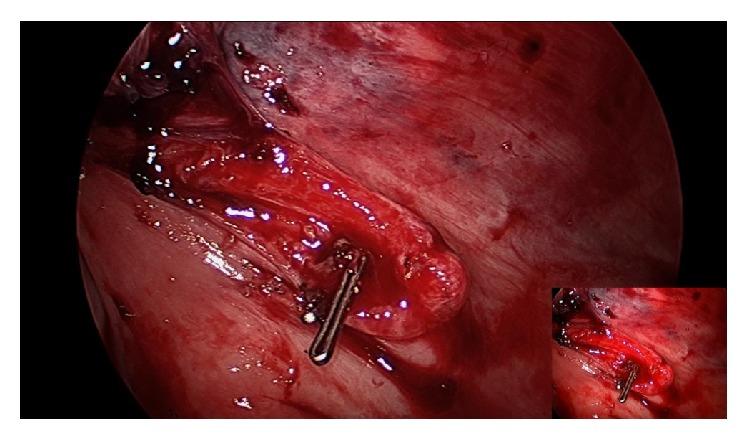
Bleeding Sampson's artery found on left inguinal hernia repair site; hemorrhage stopped with a 5 mm hemoclip.

A 17-year-old African American female, with no significant medical or surgical history, presented to the surgery clinic with bilateral inguinal masses. Physical examination confirmed the presence of bilateral indirect inguinal hernias. Following a detailed explanation of the alternatives, the patient and her mother chose an open repair. The Lichtenstein technique was used to repair both inguinal hernias and the estimated blood loss was below 5 mL. No intraoperative complication or anatomical variation was identified. Following completion of surgery at a late hour, the patient was admitted for overnight monitoring. The following day, she began complaining of severe epigastric pain. On physical exam, the abdomen was diffusely tender to palpation and slightly distended with no peritoneal signs. Vital signs were stable, and there was no orthostatic hypotension. Stat blood work showed a 5 g hemoglobin drop from preoperative hemoglobin and hematocrit values of 12.7 g/dL and 38.2% to postoperative results of 8.7 g/dL and 27.1%, respectively. Computed tomography (CT) of the abdomen and pelvis revealed a remarkable hemoperitoneum so the patient was taken to the operating room for an emergent diagnostic laparoscopy. During exploration, a patent, bleeding Sampson's artery was found on the left inguinal hernia repair site and the hemorrhage was stopped using 5 mm hemoclips (Figure 1). The estimated loss of 1 L of blood and clots was evacuated by vigorous peritoneal cavity irrigation with normal saline. The patient was discharged the following day and had an otherwise unremarkable recovery. Follow-up at 1 month and 6 months showed no hernia recurrence.

## 3. Discussion

Although the management of intra-abdominal hemorrhage after open inguinal hernia repair using a laparoscopic approach was described over ten years ago [4], bleeding from a patent Sampson's artery has been reported only once before [3]. In that report, as in this case report, the complication occurred within the first 24 hours after elective open inguinal hernia repair. This complication resulted in a significant drop in the hemoglobin count and a sizable hemoperitoneum. Careful surgical technique with attention to proper hemostasis generally obviates the need for a formal vascular intervention [3]. The round ligament is routinely ligated during hernia repair. It is not considered an essential structure, a remnant of the gubernaculum; it passes from the uterus to the deep inguinal ring where it enters the inguinal canal to attach to the connective tissue associated with the labia majora [5]. The blood supply to the upper adnexal structures is derived from the paired ovarian arteries, which originate from the aorta [3, 6]. The uterine artery, which is a branch of the internal iliac artery, supplies the uterine structures [6]. An arterial arcade formed by these vessels runs in the lateral adnexa and gives of a small arterial branch to the round ligament called Sampson's artery or the artery of the round ligament [3, 6]. Sampson's arteries are normally obliterated in postembryonic development; however they may remain patent as sources of collateral blood supply to the uterus in some patients. Although a relatively minor blood vessel, failure to properly ligate Sampson's artery can lead to significant postoperative hemorrhage [3]. LeDref et al. have reported Sampson's artery as a source of postpartum hemorrhage in patients [7]. Saraiya et al. have also reported the Sampson artery as a potential source of collateral blood supply to the uterus which interventionalist performing uterine embolization should be aware of, along with the ovarian arteries [8].

The ligation of a bleeding Sampson artery is trivial under laparoscopic visualization, and it could be performed with only one port. The laparoscopic approach also made it possible to evaluate both sides simultaneously and thoroughly, as well as the efficient and complete evacuation of the hemoperitoneum.

## 4. Conclusion

The artery of the round ligament of the uterus, also called Sampson's artery, potentially can become a source of collateral blood supply to the uterus. It can potentially cause a serious complication of hemoperitoneum in patients undergoing inguinal hernia repair, similar to complication of the artery of the round ligament of the uterus reported during hysterectomy and uterine embolization. Whether an initial laparoscopic repair instead of open technique could have averted this complication altogether remains an open question.
